# Yorkshire and Humber Less Than Full Time Trainees: What Do They Need?

**DOI:** 10.1192/bjo.2022.95

**Published:** 2022-06-20

**Authors:** Laura Shaw, Sara Davies

**Affiliations:** 1 Leeds and York Partnership NHS Foundation Trust, Leeds, United Kingdom; 2 Health Education England, Yorkshire and Humber, United Kingdom

## Abstract

**Aims:**

Due to the demand for increased flexibility of working there is an ever-increasing number of trainees working Less Than Full Time (LTFT). The Royal College of Psychiatrists supports LTFT training and careers within the specialty. However, applying for and working LTFT can be a challenging and at times overwhelming process to navigate. This project's aims are to improve written information provided to trainees when commencing LTFT training. To assess interest for a LTFT training educational event and to plan this event based on trainees preferences for content and timing.

**Methods:**

Questionnaires via SurveyMonkey were sent to higher trainees in all regions of the Yorkshire and Humber Deanery in October 2021 by the Medical Education Departments. A covering email invited those working or interested in LTFT to complete the questionnaire.

Questions assessed the need for further written information on LTFT training in the region and interest in an educational event. Trainees already working LTFT were asked what they valued most out of support already in place.

**Results:**

Of 40 trainees who responded, 100% stated when commencing LTFT training they would wish to receive more information. Respondents were asked which areas they would like included: practicalities (100% of respondents), LTFT mentor (85%), peer support (83%), weblinks to information (70%), recommended reading (53%). Those already working LTFT were asked what they had found helpful, the most common themes were mentoring, peer and supervisor support.

85% of trainees surveyed confirmed they would be interested in attending a LTFT focused educational event. Based on trainee preferences the areas to be included were job planning (78%), choosing a job (81%), clinical lead views (78%), finances (86%), Out Of Programme opportunities (61%) and emotional aspects (61%). Further questions clarified preferences for a virtual Vs face-to-face meeting and timing to maximise attendance.

**Conclusion:**

There is a need for further information to be provided to trainees on LTFT training in the region. The content has been guided by trainees and will be incorporated into the Higher Trainee Handbook.

For those training LTFT the most helpful aspect has been mentoring, peer and supervisor support. We have subsequently linked with the established mentoring scheme and those new to LTFT are matched with an established LTFT trainee. We recommend 3 monthly LTFT peer and Training Programme Director meetings continue.

There is considerable interest in an educational event focused on LTFT working. This has been organised for May 2022 and the content guided by trainee's preferences.

